# Peer Review of “Supporting Technologies for COVID-19 Prevention: Systemized Review”

**DOI:** 10.2196/38728

**Published:** 2022-05-24

**Keywords:** COVID-19, medical treatments, personal protective equipment, testing methods

*This is a peer-review report submitted for the paper "Supporting Technologies for COVID-19 Prevention: Systemized Review".*

## Review Round 1

### General Comments

The manuscript [1] talks about medical technologies during COVID-19. The review is nice to read. I could not find Table 2.

### Specific Comments

#### Major Comments

1. My main concern is that several technologies are missing, so I am not sure if the review on Google search was carried out properly. There must be definitely over 90 technologies. If you check the Federal Drug Administration (FDA) In Vitro Diagnostics, there are over 240 test kits alone. Additionally, I am not sure how you reach to 38 items from 90, or are there so many unrelated items?
2. The images in the figures, especially on company products, need actual permission from the original company or inventor. For example, the image citing reference 2 is a Britsh Broadcasting Corporation (BBC) article, but the actual image is from a hospital whose permission is needed, rather than citing BBC.
3. Several topics are outdated as of now, such as personal protective equipment. The interest in smart or green personal protective equipment has declined dramatically as vaccination has picked up. Therefore, the text needs to be aligned with current needs, such as low-temperature storage technologies to store vaccines, etc. The ventilators section is interesting, but such images have been shown before in many places. As such, it will be difficult to garner readership based on the sections.
4. Several points are being repeated throughout the manuscript, such as lack of manpower and resources. The flow of the text could be more fast paced by removing general statements and sticking to facts only.
5. New and interesting topics could be added based on the current status of the pandemic, such as technologies centering around vaccination or at-home testing.

## Abbreviations

BBC: British Broadcasting Corporation
FDA: Federal Drug Administration
